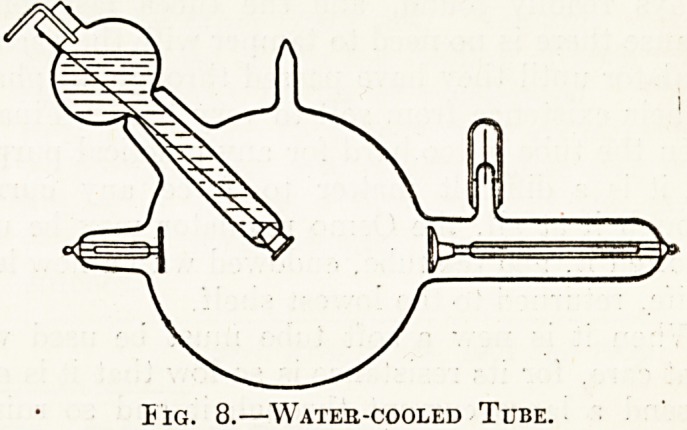# The X-Rays

**Published:** 1912-08-03

**Authors:** Alfred C. Norman

**Affiliations:** House Surgeon at Sunderland County Eye Infirmary.


Fig. 1.—Myeloma, Superior Extremity of Tibia.

Fig. 2.—Tuberculous Focus, Superior Extremity of Tibia.

Fig. 3.—Fracture, Olecranon Process.

Fig. 4.—Fracture Dislocation, Scaphoid Bone of Foot.

Fig. 5.—Fracture Separation, Radial Epiphysis.

Fig. 6.—Cancellous Osteoma, Upper Part of Humerus.

Radiographs made by Dr. A. C. Norman in 1909 with a 12-inch Coil and Centrifugal Mercury Interrupter.

### ELECTRICITY IN MODERN MEDICINE.*

### XV.—The X-Rays

By ALFRED C. NORMAN, M.D. Edin., House Surgeon at Sunderland County Eye Infirmary.

Speaking generally, the more a tube costs the longer will it last and the more satisfaction will it give during the whole of its working life. The quantity of *x*-rays discharged at a given moment depends not upon the size of the tube but upon the amount of current passing through it. A large tube will stand, without undergoing detrimental changes, more current than a small one, because it is not so readily overheated; also because it contains more residual gas for the same degree of exhaustion, hence any gas that may be released from the anticathode has a proportionately smaller effect on the vacuum. *X*-ray tubes can be obtained in various sizes from 3 to 8 inches diameter of the main bulb, but it will be found good economy in the long run to purchase the largest ones because their increased length of life justifies their first cost, they give much less trouble owing to their vacuum requiring so little attention, and they will stand much larger currents,

Fig. 7.—Bauer Tube.

hence considerably shorter exposures can be given when radiographs are being taken.

The science of coil building has so far outstripped that of tube making that at present there is no tube obtainable that will stand for more than a very few seconds the full discharge from a modern induction coil. This is mainly due to the immense heat generated in the anticathode as a result of its bombardment by the stream of cathode rays, and to minimise as far as possible this heating effect all the best tubes are now constructed with "heavy" anticathodes. A heavy anticathode—*i.e.* one made in the form of a solid block of metal—takes some time to become dangerously hot, and will stand a large current without risk of melting, but, unfortunately, the metals which make satisfactory anticathodes are very costly, hence the price of these tubes is very high. Platinum is undoubtedly the best metal for the surface of the anticathode; it has a high melting-point and it gives rise to *x*-rays of excellent quality, but, unfortunately, it has two drawbacks: its enormous cost; and the property of absorbing a larger amount of gas than almost any other metal and of liberating this gas when it becomes hot. For these reasons it would be out of the question to construct a "heavy" anticathode of solid platinum, so the best tubes are now made with a heavy block of nickel or copper faced by a thin disc of platinum, and very satisfactory they are. In addition to the "heavy" anticathode, different makers adopt different devices for cooling the tube. In all cases the metal stem supporting the anticathode is made as solid as possible with a view to radiating away heat from the latter; in the Gundelach model there is a tube of metal surrounding the anticathode and its stem which forms a very efficient radiator (fig. 2, p. 350), and in the Bauer tube (fig. 7) there is a copper radiator which fulfils the same purpose.

Another form of cooling device is seen in the "water-cooled" tube (fig. 8). In this type the anticathode and its stem are hollow and must be kept full of water while the tube is working. Some of these tubes will stand a very large current, but they have the disadvantage that they can only be used in certain positions, otherwise the water would not be

Fig. 8.—Water-cooled Tube.

in contact with the anticathode and the latter would be quickly burnt out.

The cathodes of all tubes are made of aluminium because this metal has less tendency than most to disintegrate under the stress of discharging the stream of negative electrons. Platinum, on the other hand, disintegrates very rapidly when it functions as a cathode, and the reason why reverse current does so much harm is that the platinum-faced anticathode, functioning as a cathode to the reverse current, becomes disintegrated into minute particles which rapidly absorb the free gas in the tube.

There are many makes of tube on the market that satisfactorily fulfil all the conditions we have just considered. Bauer's are excellent, and so are Müller's; but it is advisable to adopt one pattern and stick to it, so that one may become familiar with its idiosyncrasies. The writer invariably uses 8-inch Gundelach "heavy" anticathode tubes with Osmo regulation, and he could not wish for anything better. These tubes cost about £3 15s. each, and at least three should be kept in readiness. Excellent results can be obtained with small, light tubes, such as fig. 1, page 350, costing less than £1, but their normal current is very small, hence long exposures have to be given, and the tubes require constant watching.

*X*-ray tubes should have a special cupboard to

* Previous articles appeared on Nov. 11, 25, Dec. 9, 30, Jan. 13, 27, Feb. 17, March 9, 30, April 20, May 4, 25, June 8, and July 6.

themselves, which should be warm and dry and should be kept rigidly free from dust. The shelves should be furnished with padded holes large enough to pass the cathode stem in order that the main bulbs of the tubes may be supported by the circumference of the hole in the shelf.

There should be three shelves in the cupboard, labelled "hard," "medium," and "soft," respectively. A new tube should always be ordered from the makers in a soft condition, and should be kept on the "soft" shelf as long as it is suitable for purposes requiring rays of low penetration—*e.g.* radiographing fingers and toes and certain therapeutic applications. As it gets harder it should be moved to the "medium" shelf, and may then be used for many therapeutic applications and for radiographing knees, shoulders, and the like. When the rays become too penetrating for these purposes the tube should be moved to the "hard" shelf and be used for making radiographs of the skull and other parts of the body that require rays of high penetrative power. By adopting the above procedure a tube suitable for the work in hand is always readily found, and the tubes last longer because there is no need to tamper with the vacuum regulator until they have passed through all phases of their existence from soft to very hard. Finally, when the tube is too hard for any practical purpose and it is a difficult matter to force any current through it at all, the Osmo regulator may be used to soften it, and the tube, endowed with a new lease of life, returned to the lowest shelf.

When it is new a soft tube must be used with great care, for its resistance is so low that it is easy to send a large current through it and so ruin it in a few seconds. A valve tube or spark-gap should always be used in series with a new tube to suppress reverse current. Reverse current is always of lower voltage than normal current, consequently a fairly hard tube will keep a good deal of it back; but a soft tube, on account of its low resistance, will pass reverse almost as easily as normal current, hence the need for a valve tube.

For every tube there is a certain current (termed by Mr. Schall the "normal" current) which will keep the vacuum practically constant. It is that quantity of current which will liberate from the anticathode just as much gas as is being occluded in the glass walls of the tube and no more. Fortunately, we have in the milliampere-meter an instrument by which this current can be exactly determined. The milliampere-meter is connected in series with the tube in the secondary circuit, and the rise or fall of the current, shown by the needle of the meter, affords a valuable indication of what is taking place inside the tube. Suppose, for instance, that the current gradually increases while the tube is working; we know at once that we have been sending too much current through the tube, and that this has lowered its resistance, so we cut down the current as soon as possible. On the other hand, if the current steadily diminish it is an indication that the resistance of the tube is increasing, and we must increase the current accordingly. If, however, the needle of the milliampere-meter remains stationary we are satisfied that we have hit off the "normal" current for that particular tube, and that there is no need for further interference.

The introduction of the milliampere-meter has greatly simplified $x$-ray work, and no beginner should attempt to use an $x$-ray tube without the aid of this valuable piece of apparatus. It affords a most delicate indication that the tube is becoming soft or hard and this at a much earlier stage than is possible by merely observing the appearance of the tube. Every radiographer knows, of course, that an extremely soft tube gives out a very yellow fluorescence, that its discharge is ominously silent, and that there is a blue beam of light round the cathode; but by the time the untrained observer has noticed these changes the vacuum has probably been reduced to nearly the Geissler degree, and the tube is more or less permanently damaged.

If a tube be properly treated from the outset it becomes seasoned—that is to say, the gases dissolved in the anticathode are gradually used up, and this means, of course, that the "normal" current of the tube becomes greater as the tube gets older. For instance, the "normal" current of a new Gundelach heavy anode tube may not be more than .5 milliampere, but when it becomes seasoned it may be safely used with a current of 3 milliamperes, or even more.

Of course, it is not always possible to use a tube at its normal current. In giving therapeutic applications, for instance, very small doses are sometimes required, and then the tube needs constant watching; and if it is seen to be getting too hard it should be given a very powerful current for a few seconds to reduce its vacuum. In rapid radiography, on the other hand, it is often necessary to work a tube far in excess of its normal current, and here again the damage is not serious if due precautions be taken. The amount of heat produced in a tube is, roughly, dependent upon the time and the milliamperage; but it takes a certain time before the anticathode becomes appreciably hot—hence a tube will stand an enormous current if it be applied for a small fraction of a second, simply because the current is switched off before it has time to heat up the anticathode. It is quite common in instantaneous radiography to send more than 100 milliamperes through a tube for one-thirtieth of a second, and this does not damage the tube nearly so much as a current of, say, 10 milliamperes for several seconds.

If a tube should accidentally be allowed to become too soft it must be set aside for a prolonged rest, and this will give some of the gas time to become re-absorbed by the metal. It should then be worked for some time on a current much below its normal, and this will probably complete the occlusion of the superfluous gas; but in some cases nothing but re-exhaustion by the makers will restore the tube to a suitable degree of vacuum.

The radiographs in the special plate (figs. 1 to 6) were all taken with a Gundelach "heavy" anticathode tube. They will be referred to again in the sections on exposure and penetration.

## Figures and Tables

**Fig. 1. f1:**
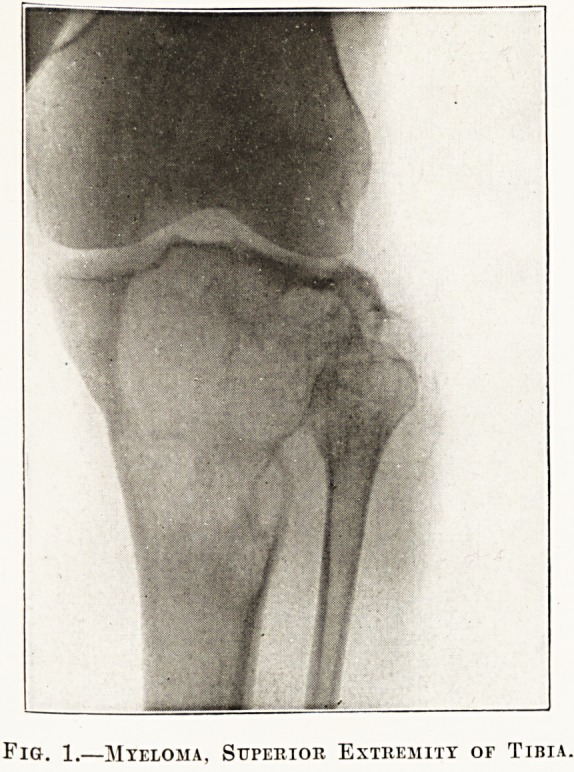


**Fig. 2. f2:**
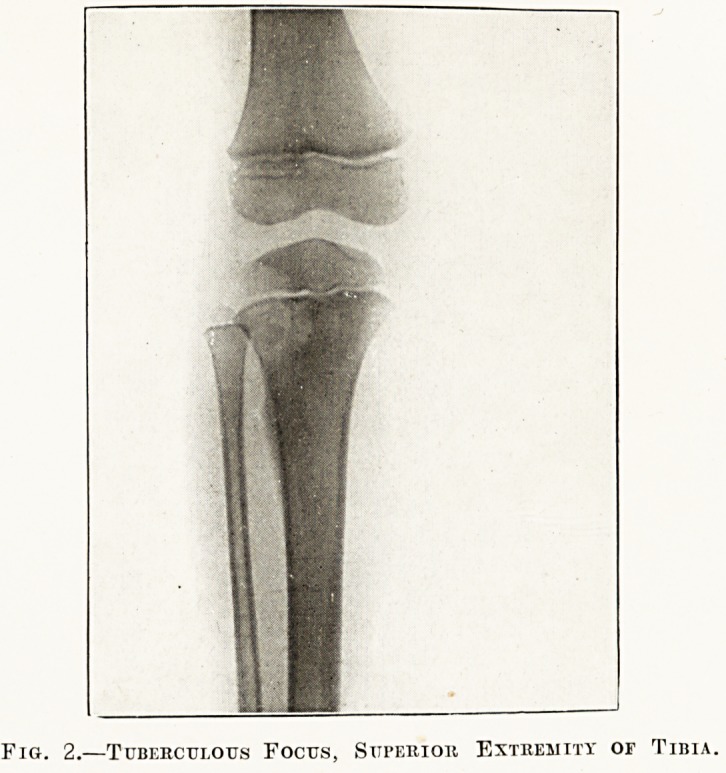


**Fig. 3. f3:**
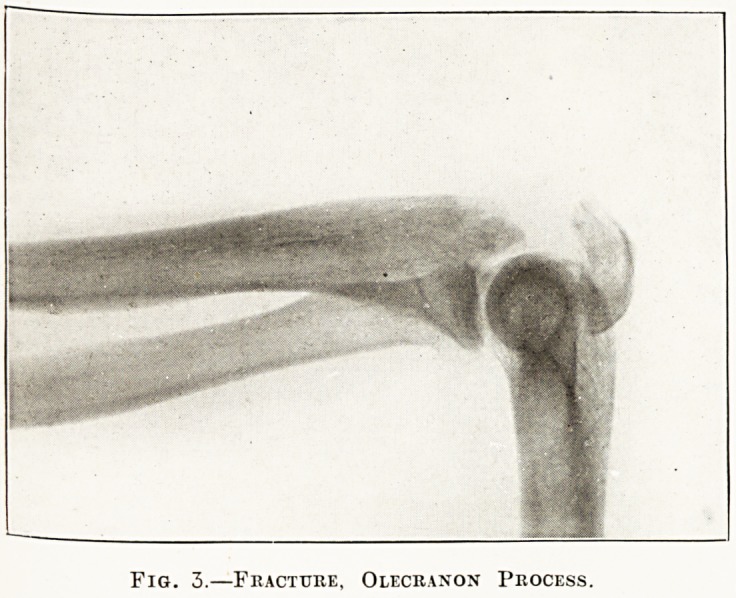


**Fig. 4. f4:**
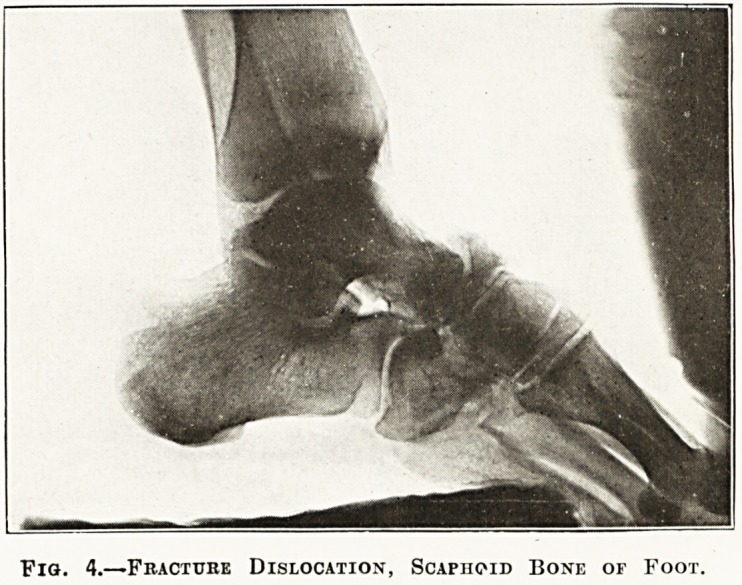


**Fig. 5. f5:**
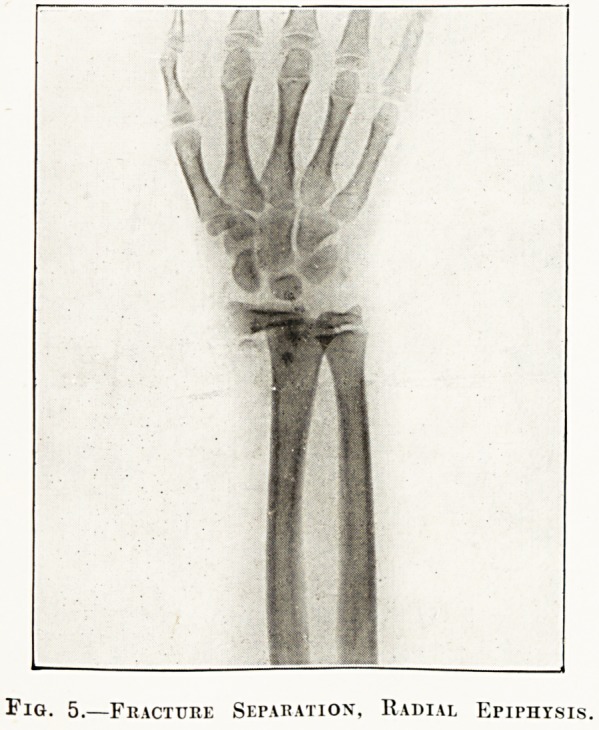


**Fig. 6. f6:**
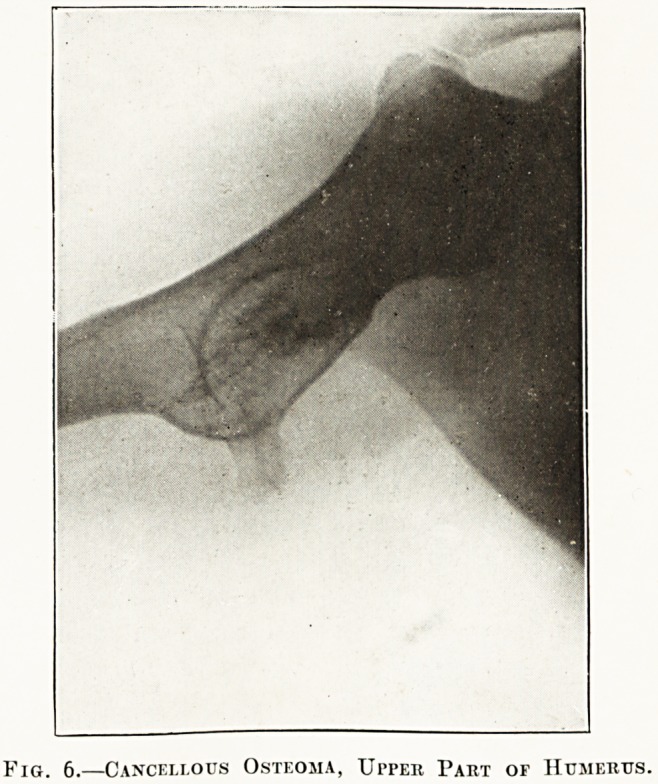


**Fig. 7. f7:**
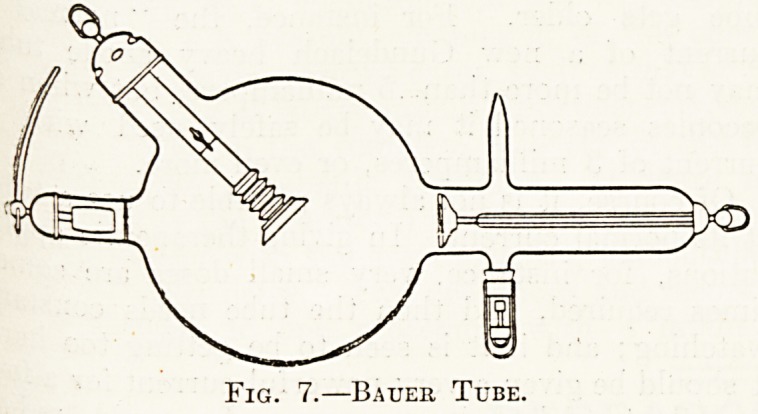


**Fig. 8. f8:**